# Impaired olfactory neurogenesis affects the performance of olfactory-guided behavior in aged female opossums

**DOI:** 10.1038/s41598-021-83834-5

**Published:** 2021-02-24

**Authors:** Beata Tepper, Paulina Koguc-Sobolewska, Katarzyna Jaslan, Krzysztof Turlejski, Katarzyna Bartkowska, Ruzanna Djavadian

**Affiliations:** 1 Laboratory of Calcium Binding Proteins, Nencki Institute of Experimental Biology Polish Academy of Sciences, Warsaw, Poland; 2 Faculty of Biology and Environmental Sciences, Cardinal Stefan Wyszynski University in Warsaw, Warsaw, Poland

**Keywords:** Neural stem cells, Learning and memory

## Abstract

Increasing evidence has indicated that adult neurogenesis contributes to brain plasticity, although function of new neurons is still under debate. In opossums, we performed an olfactory-guided behavior task and examined the association between olfactory discrimination-guided behavior and adult neurogenesis in the olfactory bulb (OB). We found that young and aged opossums of either sex learned to find food buried in litter using olfactory cues. However, aged females required more time to find food compared to aged males and young opossums of both sexes. The levels of doublecortin, that is used as a marker for immature neurons, were the lowest in the OB of aged female opossums. Another protein, HuD that is associated with learning and memory, was detected in all layers of the OB, except the granule cell layer, where a high density of DCX cells was detected. The level of HuD was higher in aged opossums compared to young opossums. This indicates that HuD is involved in plasticity and negatively regulates olfactory perception. The majority of 2-year-old female opossums are in the post-reproductive age but males of this age are still sexually active. We suggest that in aged female opossums neural plasticity induced by adult neurogenesis decreases due to their hormonal decline.

## Introduction

Olfaction is one of essential senses that plays an important role throughout life of animals. Activation of the olfactory sensory pathway starts with stimulation of olfactory receptor neurons and then this information reaches the olfactory bulb (OB). The OB is the first brain structure in the odor information coding system. Morphometric analysis of the OB has shown that the volume of the OB measured in relation to the whole brain differs within mammalian species. For example, of the three species, human, dog and goat, the greatest volume of the OB (0.31%) was noted in dog, and the smallest (0.01%) in human^1^. However, it does not mean that humans olfaction is worse. The number of OB neurons in human is the same as in other mammals. Moreover, humans can detected some odors that dogs cannot^2^.

The OB contains morphologically different types of neurons. Granule cells, which are the largest population of neurons in the OB have a unique property. They are constantly replaced with newly generated neurons that are generated in the subventricular zone of the lateral ventricle (SVZ) and then migrate through the rostral migratory stream (RMS) to the OB^3^. Newly generated neurons are present in the OB of many mammalian species. However, the existence of adult neurogenesis in humans remains controversial^4,5^. Recently, using single-cell analysis of olfactory cells Durante and colleagues have demonstrated that stem cells and neurons are produced in the neuroepithelium of the human OB^6^.

Research on marsupial species provides evidence that adult neurogenesis continues in the SVZ and dentate gyrus throughout life and the rate of neurogenesis declines with age^7^. Moreover, pharmacological interventions targeting adult neurogenesis revealed that the increased number of newly born neurons does not affect the behavior of opossum in the olfactory task, while the decreased number impairs olfactory perception^8^. Our recent findings have shown that there is a correlation between individual differences in the level of doublecortin (DCX), that is a marker for immature neurons, and performance in the Morris water maze test^9^. Notably, opossums with high levels of DCX in the hippocampus displayed better performance in the water maze test.

Aging is associated with a decline in olfactory perception^10^. Odor perception is a special process that is closely linked to memory and plasticity. HuD, is a RNA-binding protein and mainly expressed in projecting neurons of several brain regions, which along with other proteins controls synaptic plasticity^11–13^. Changes in HuD expression are associated with hippocampal spatial learning and memory processes^14,15^.

To test whether there are differences in odor perception between sex and age in opossums, we performed olfactory-guided behavioral test in young and aged animals of both sexes. We hypothesized that olfactory perception in aged opossums should decline due to reduced adult neurogenesis. Next, we asked whether expression of HuD protein is associated with learning and memory in relation to olfactory perception.

## Results

### Body weight

**Figure 1:**
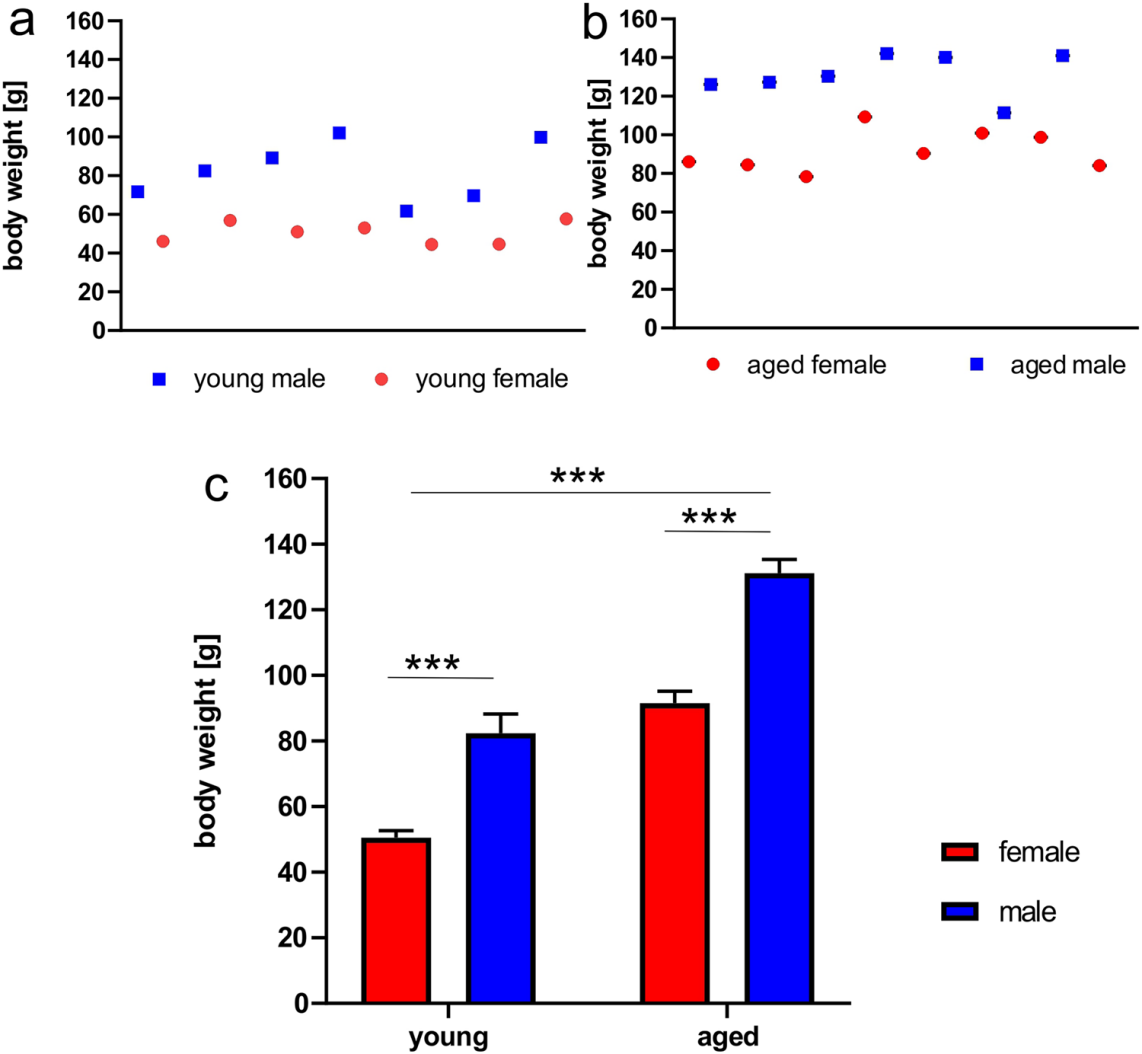
Opossums body weight. Body weight of young females and males (**a**) and aged females and males (**b**). Analysis of body weight measurements in young and aged opossums of both sexes. In (**c**) ***indicates statistical significant difference *p* < 0.0001.

The experiments were carried out on young, 5-month-old and aged, 24-month-old opossums of both sexes. The body weight of the opossums was monitored. The animals were weighed twice, before performing the behavioral test and immediately after the last test. Sexual dimorphism of body weight was clearly seen at 5-month-old opossums (Fig. 1a), namely female opossums had a lower body weight (50.5 ± 2.1 g) than male opossums (82.3 ± 5.8 g). Aged male and female opossums also had different body weight (Fig. 1b). The body weight of aged female was 91.5 ± 3.7 g, while male opossums were weighed on average 131.2 ± 4.1 g. We performed statistical analysis using a two-way ANOVA, with age and sex as independent variables. Statistical analysis showed that the body weight of opossums differed significantly in groups (Fig. 1c). There were significant main effects of age (young opossums weighed less than aged opossums, F_(1,25)_ = 116.9, *p* < 0.0001) and sex (males were heavier than females, F_(1,25)_ = 74.02, *p* < 0.0001). After the last test opossums were again weighed. Restriction to food led to a maximum of 15% body weight loss in a few opossums, while the average body weight loss was about 10% of their initial body weight.

### Behavioral olfactory discrimination test

**Figure 2:**
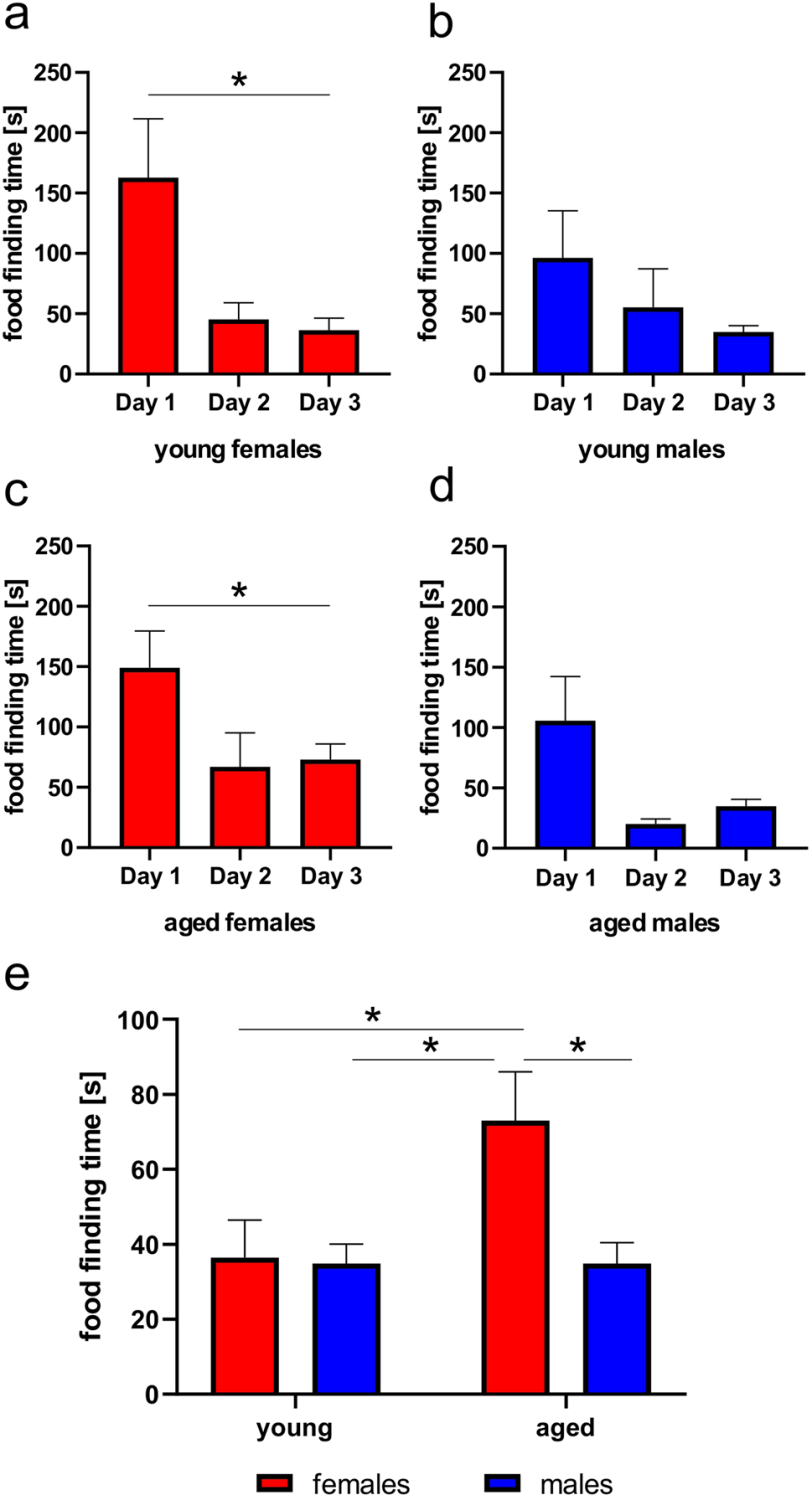
Performance of opossums in olfactory-guided test. (**a**–**d**) Time for food location during the first trail of day 1, the first trail of day 2 and testing day 3. Quantification of time to digging in the place with orange odorant where a piece of orange was previously offered.

To study learning ability of young and aged opossums using olfactory cues, we performed the following olfactory discrimination test in a three-day paradigm. The first 2 days were training days with 4 trials for each opossum, during which opossums learned to find food in a new environment that was associated with one of the two different odorants. Odorants were lemon and orange that contain the same molecule called limonene. In fruits this molecule has different structure and animals detect as different odorants. A piece of orange was buried in sawdust in the same place where the orange scent was introduced. Opossums were restricted from food 24 h prior to perform discrimination test. During the first trail on the first day opossums sniffed, ran, dug and climbed the metal wires of the cage cover. In general, all tested opossums investigated the new environment and the majority of them found by chance food buried in the sawdust (Fig. 2a–d). However, during the first trial lasting 5 min three young females and one male did not find buried food, while only one aged female and one male could not find food. Although both young and aged females spent more time to find food than male opossums (young females 162.9 ± 48.7 s vs. young males 96.4 ± 38.8 s, and aged females 149.0 ± 30.4 s vs. aged males 105.6 ± 36.6 s), the statistical analysis using a two-way ANOVA showed that there was no significant difference between young female and male groups. Using paired t test, significant differences were observed in young and aged female groups between the first trial on day 1 and the first trial on day 2 (Fig. 2a,c). Young (t = 2.85, df = 6, *p* = 0.02) and aged (t = 3.24, df = 7, *p* = 0.01) females shortened time to find food, while both young (t = 1.82, df = 6, *p* = 0.11) and aged (t = 2.32, df = 6, *p* = 0.059) males found food in a short time after being placed in a new environment (Fig. 2b,d).

On the third day, a probe test was performed without food reward and the parameters were measured allowing to estimate the level of learning. Analyzing time to the first digging in the scented place where food with the same odor was previously offered, we found that aged female opossums needed more time to start digging (Fig. 2e). The statistical analysis of these data was carried out using a two-way analysis of variance, where independent variables were sex and age. A significant difference was observed in sex (F_(1,25)_ = 4.39, *p* = 0.04), while the age factor (F_(1,25)_ = 3.72, *p* = 0.06) and interaction of variables (F_(1,25)_ = 3.72, *p* = 0.06) were just on the verge of being significant. Tukey’s multiple comparison post hoc test showed significant difference between aged female opossums and all other groups.

### Brain and OB weight

**Figure 3:**
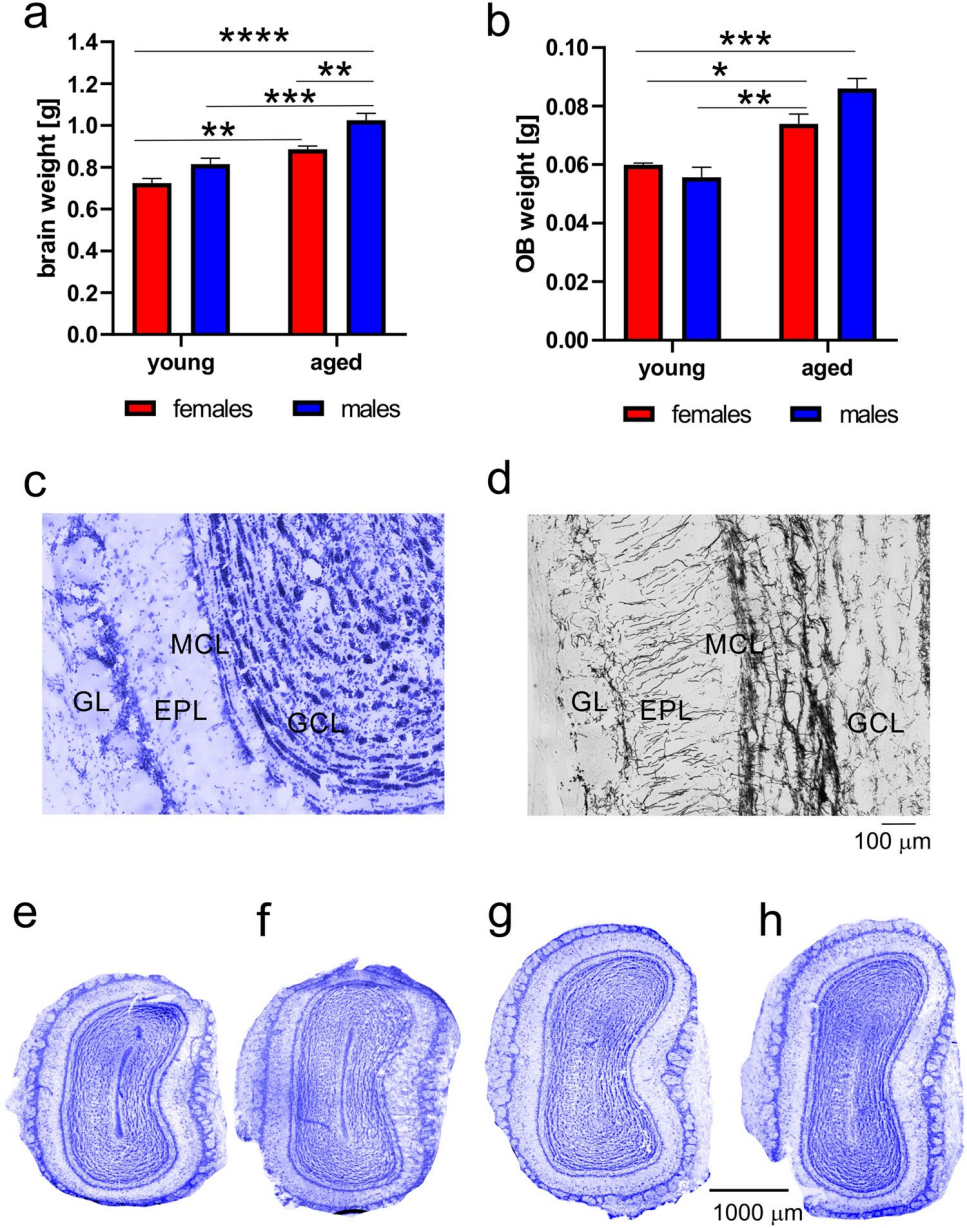
Brain and olfactory bulb (OB) weights of young and aged opossums, and cyto- and myelo-architectonic characteristics of the OB. (**a**,**b**) Quantification of brain (**a**) and olfactory (**b**) weight measures in young and aged opossums of both sexes. (**c**,**d**) Cyto- (**c**) and myelo- (**d**) architectonic properties of the OB. (**e**–**h**) Coronal Nissl-stained OB sections in young female (**e**), young male (**f**), aged female (**g**) and aged male (**h**) opossums. *GL* glomerular layer, *EPL* external plexiform layer, *MCL* mitral cell layer, *GCL* granule cell layer. In (**a**,**b**) *p < 0.01; **p < 0.001; ***p < 0.0001, ****p < 0.00001. The scale bar in (**d**) refers to (**c**), and the scale bar in (**h**) refers to (**e**–**h**).

The OB of the opossum is relatively large compared to the same size of the brain’s eutherian species. To estimate OB weight in relation to the brain, brains of opossums used for Western blot analysis were weighed and OB were separated and also weighed (Fig. 3a,b). The brain weight of young opossums (female 0.73 ± 0.021 g and male 0.81 ± 0.027 g) was lower than aged opossums (female 0.88 ± 0.015 g and males 1.02 ± 0.032 g). The two-way ANOVA analysis revealed that there were significant main effects between sex (F_(1,12)_ = 21.33, *p* = 0.0006) and age (F_(1,12)_ = 55.85, *p* < 0.0001), but no significant interaction between them (F_(1,12)_ = 0.88, *p* = 0.36). Interestingly, the two-way ANOVA analysis for OB showed significant effect in age (F_(1,12)_ = 53.53, *p* < 0.0001) but not in sex (F_(1,12)_ = 1.642, *p* = 0.22), while a statistical significant interaction was between sex and age (F_(1,12)_ = 7.22, *p* = 0.01). Next, we calculated the percentage of OB weight in relation to the brain weight. In aged opossums of both sexes it was 8.3% while in young opossums it was 8.1% and 7.1% in females and males respectively.

The OB of the opossums has a layered organization like other mammalian species. All 4 layers, the glomerular layer, the external plexiform layer, the mitral cell layer and the granule cell layer were visible in Nissl-stained (Fig. 3c) and myelin-stained sections (Fig. 3d).

Given that the OB weight was different for young and aged opossums, we analyzed Nissl-stained images. Coronal sections illustrated in Fig. 3e–h were taken at the same level of OB. This figure illustrates that Nissl-stained sections in different groups were varied by size (Fig. 3e–h).

### Neurogenesis and plasticity in the OB

**Figure 4:**
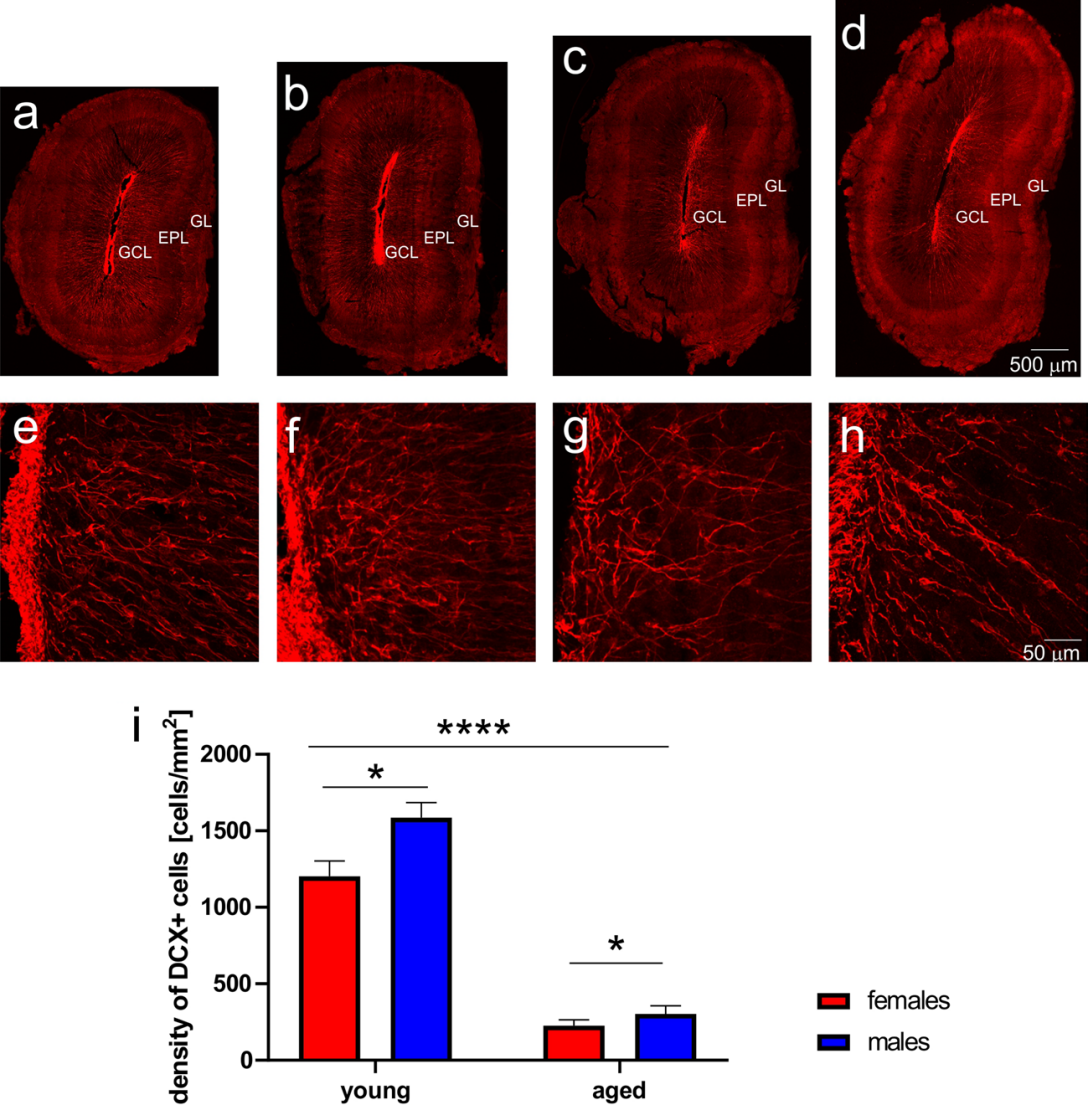
Localization of doublecortin (DCX) in the olfactory bulb (OB) of opossums. (**a**–**d**) DCX labeled neurons in the young female (**a**), young male (**b**), aged female (**c**) and aged male (**d**) OB. (**e**–**h**) High magnification images taken from images presented in (**a**–**d**), showing localization of DCX labeled cells in the granule layer (**e**–**h**). (**i**) Quantification of DCX immunolabeled cell density in the OB of young and aged oposumms. *GL* glomerular layer, *EPL* external plexiform layer, *GCL* granule cell layer. The scale bar in (**d**) refers to (**a**–**d**), and the scale bar in (**h**) refers to (**e**–**h**). In (**i**) *p = 0.018, ****p < 0.0001.

In adult brain, DCX is expressed in immature neurons, and we used it as a marker for neurogenesis. To address distribution of DCX expressing neurons in the OB during aging, brain sections were immunostained with antibody against DCX. In young opossums of both sexes DCX immunopositive cells were located in all layers of the OB with the high density in the granule cell layer (Fig. 4a,b,e,f). A somewhat different pattern was observed in aged opossums. DCX immunostained cells were observed in all layers apart from the glomerular layer (Fig. 4c,d). Similarly, the high density was observed in the granule layer (Fig. 4g,h). However, no cell was immunolabeled with DCX in the glomerular layer of aged opossums. We estimated the number of cells in the OB. The two-way ANOVA showed a very strong difference between young and aged opossums (F_(1,8)_ = 211.2, *p* < 0.0001). There was also a significant main effect of sex (F_(1,8)_ = 8.698, *p* = 0.018). The highest number of DCX cells was observed in young male opossums (Fig. 4i).

**Figure 5:**
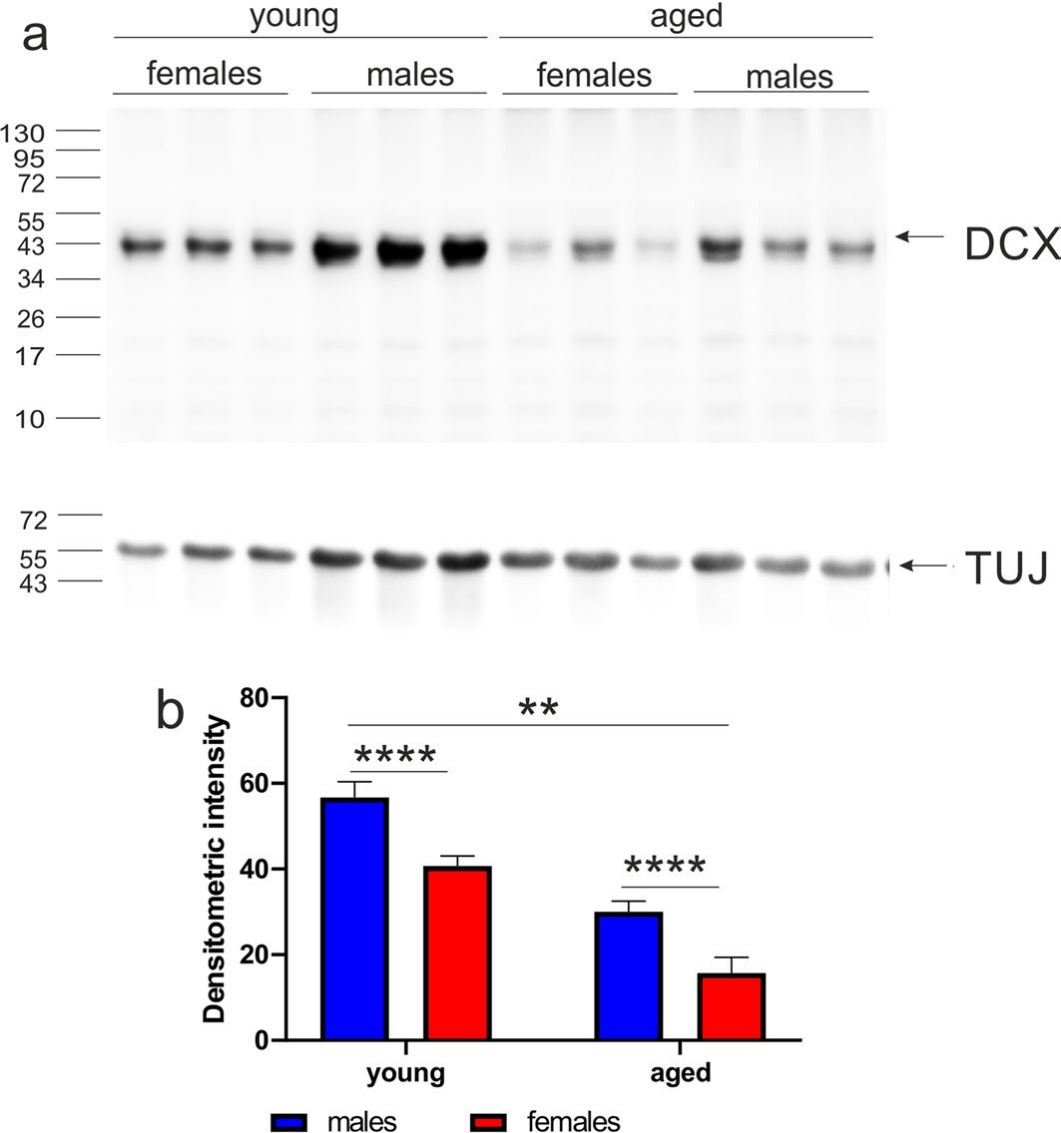
Western blot analysis of doublecortin (DCX) in the olfactory bulb (OB) of opossums. (**a**) Each immunolabeled band shows the amount of DCX in the OB of an individual opossum. Beta-III tubulin (TUJ) was used as a loading control (Supplementary Fig. S1a). (**b**) Quantification of DCX levels from Western blots. **indicates statistical significant difference *p* < 0.001, and ****indicates *p* < 0. 00001.

The density of DCX-immunoreactive cells decreased with aging (Fig. 4i). To quantify this, we performed Western blot analysis evaluating the level of DCX protein in the OB homogenates. Western blotting showed that the strongest labeled bands (DCX protein) were detected in young male opossums (Fig. 5a). The level of DCX in different groups of opossums was analyzed in relation to the reference beta-III tubulin (TUJ) protein (Fig. 5). We found that both young and aged males had a high level of DCX in the OB compared to females of the same age. The lowest level of DCX was observed in aged female opossums (Fig. 5b). These differences were statistically significant. The two-way ANOVA detected differences in both variables, age (F_(1,8)_ = 23.59, *p* < 0.001) and sex (F_(1,8)_ = 68.45, *p* < 0.0001).

**Figure 6:**
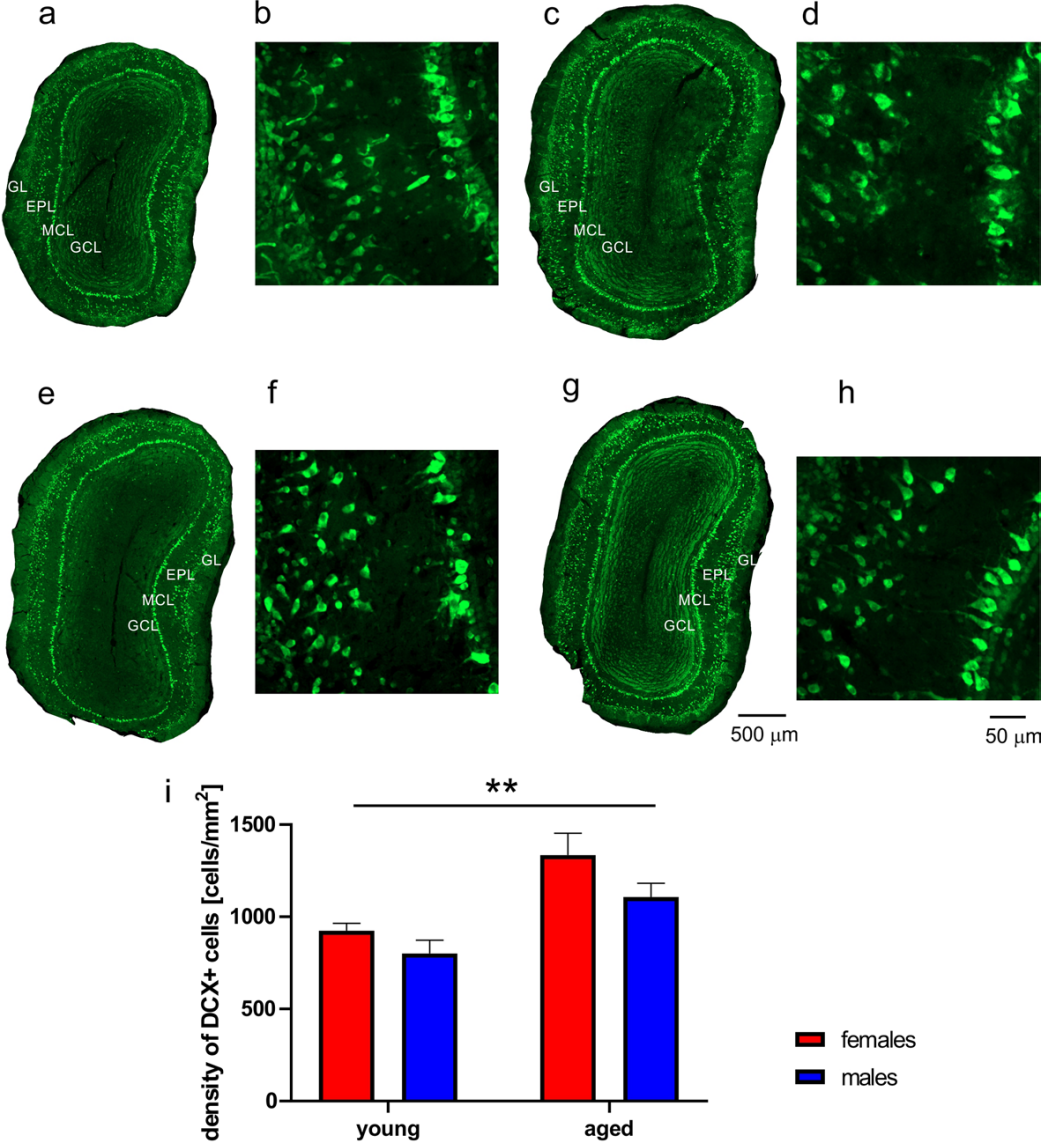
Localization of HuD in the olfactory bulb (OB) of opossums. (**a**–**h**) HuD immunostained neurons in the OB of young females (**a**,**b**), young males (**c**,**d**), aged females (**e**,**f**) and aged males (**g**,**h**). (**b**,**d**,**f**,**h**) Higher magnification images from (**a**,**c**,**e**,**g**) respectively. *GL* glomerular layer, *EPL* external plexiform layer, *MCL* mitral cell layer, *GCL* granule cell layer. The scale bar in (**h**) refers to (**b**,**d**,**f**,**h**) and the scale bar in (**g**) refers to (**a**,**c**,**e**,**g**). In (**i**) **indicates *p* = 0.002.

Adult neurogenesis is associated with plasticity. To examine whether new born cells incorporated into neuronal circuits could contribute to learning and memory processes in opossums, we studied expression of HuD in the OB. Localization of HuD protein was defined by immunohistological technique. For this purpose brain sections representing the OB were immunostained with antibody directed against HuD. Numerous HuD-immunopositive cells were placed in the OB of both young and aged opossums (Fig. 6a,c,e,g) and limited to only upper layers. An intensive staining of HuD was present in the mitral cell layer, the external plexiform layer and the glomerular layer, while this labeling was almost absent in the granule cell layer (Fig. 6b,d,f,h). To estimate the number of HuD labeled cells in young and aged opossums we calculated their density. We found a significant main effect of age (F_(1,8)_ = 19.24, *p* = 0.002), but no significant effect of sex (F_(1,8)_ = 4.611, *p* = 0.064). The number was higher in aged opossums of both sexes with more neurons in females compared with young animals (Fig. 6i).

**Figure 7:**
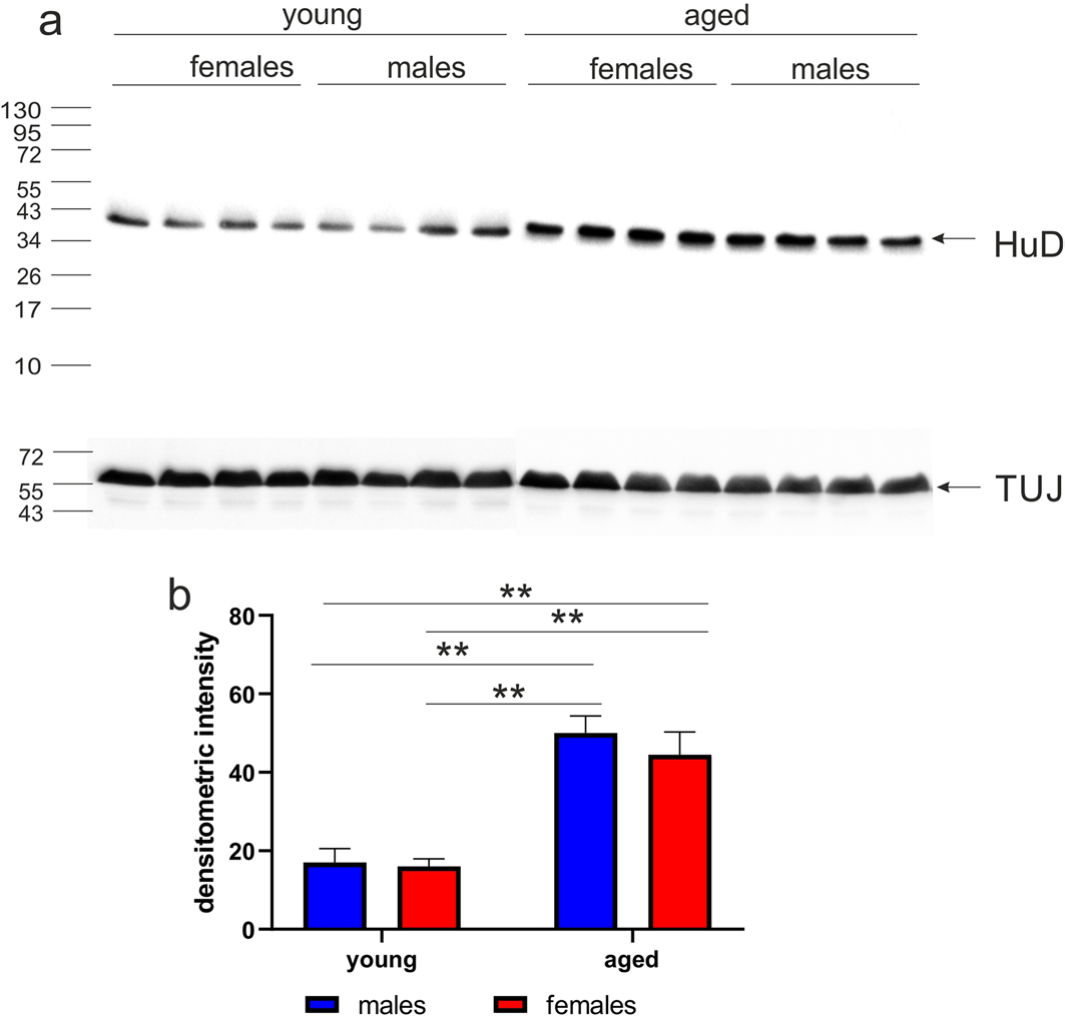
The level of HuD protein in the olfactory bulb (OB) of opossums. (**a**) Each labeled band in Western blot shows the amount of HuD in an individual opossum OB. Beta-III tubulin (TUJ) was used as a loading control (Supplementary Fig. S2a). (**b**) Quantification of HuD from Western blots. **Indicates *p* < 0.001.

We next defined the amount of HuD protein by Western blot analysis. The level of HuD expression in the OB was different for young and aged opossums (Fig. 7a). The HuD protein was lower in young opossums than in aged animals of both sexes (Fig. 7b). A two-way ANOVA revealed that there was a strong difference in age (F_(1,14)_ = 40.07, *p* < 0.0001), however no difference in sex.

## Discussion

Here, we focused on providing the olfactory discrimination test to assess learning and memory in young and aged opossums of both sexes. We found that during training days opossums using olfactory cues immediately learned to find rewarded food. On the third day, the test was performed without food and measurement of time when the opossum started to dig the place where previously rewarded food was buried showed that all groups except aged females had approximately similar time. These changes were associated with the level of DCX expressing cells in the OB. Notably, of all groups, aged females had the lowest level of DCX and the worst performance during the test day of the behavioral task. Furthermore, behavioral changes were not correlated with the level of HuD protein. The expression of HuD was lower in the OB of young opossums than in aged opossums of both sexes.

### Learning and memory using olfactory cues

We performed a simple odor-discrimination task on young and aged opossums of both sexes. A day before performing the test opossums were restricted for food. Therefore, they were hungry and motivated to find food in the new environment. Before conducting this experiment, we observed behavior of several full-fed opossums in a new environment. We found that they were just running around and exploring the new cage, sometimes sniffing intensely, but none of them dug to look for food. However, when the experimental hungry opossums were placed in the new testing cage first time, they behaved differently. After a short exploration of the new environment, most of the animals started to sniff and dig sawdust and found a piece of buried orange. On the second day of experiment, when the opossums were placed in the testing cage, they immediately began searching for food. All of them learned that the new cage is associated with food. Using olfactory cues opossums found food, although some opossums took a long time to succeed.

In rodents, behavioral tasks with olfactory stimuli were used to study the mechanisms of olfactory perception, learning and memory. Rats quickly learned to distinguish between odors to avoid aversive taste^16^. Mice also learned to perform the odor discrimination task^17^. A number of papers have shown age-related differences in learning and memory that are associated with olfaction^18–21^. However, some studies reported contradictory results. For example, Kraemer and Apfelbach reported that male Wistar rats of various ages, including old rats, showed no impairment in learning ability of odor discrimination tasks^22^. They suggested that age-related deficits in learning and memory are linked to stress and other factors, and proposed a long-lasting procedure of animals handling before the experiment. Another explanation of these data is that the chosen test was inappropriate for testing young or old animals^23,24^. We believe that hormones, including stress hormones can influence on behavioral test. Most of experiments investigating olfactory perception in rodents were done on males. Our results indicate that aged female opossums had a different performance in behavioral test than males. Two years old female opossums are in post-reproductive age, while males of the same age are sexually active and can produce offspring. We suggest that among others, hormonal changes could worsen performance aged female opossums in olfactory-guided test. Another paper has shown that responses in OB glomeruli neurons, recorded as neurotransmitter release to different odor applications, did not change in 6–24 month old mice^25^. However, they do not exclude that olfactory impairment during aging may occur in other olfactory brain structures.

More recent data also demonstrated reduced ability of discrimination between odorants having similar structure in aged F344 rats^26^. This impairment of olfactory discrimination in aged rats most probably is due to senescence, which confirms our data. We also used two odorants of similar chemical structure, orange and lemon. To avoid additional smell, we used a piece of orange that was buried in the sawdust, always in the same place where the orange oil drops were applied. Two-year-old female opossums needed more time to start digging in the orange-scented spot during the test day. This indicates that olfaction declines in normal aging.

### Adult neurogenesis and plasticity in the OB

Opossums are born immature, weighing only 110 mg. After birth the opossum attaches to the mother’s nipple and spends the next four weeks there. In our colony, opossums are separated from their mothers at 2 months of age, and then each litter is kept in a separate cage for the next 2 months. Opossums are solitary animals and are individually housed in separate cages from the age of 4 month. Our experiments were performed on young 5-month-old and aged 24-month-old opossums. Opossums’ OBs are quite large, their relative weight is 7–8% of the whole brain. In various species of mice, this proportion is up to 5%^27^. Interestingly, in all these species of mice, the OB size is increased during aging. For example, in C57BL/6J the OB weight was twice more in 300 days of age mice than in 30 days old mice. Also in rats, the OB total volume was increased by approximately 47% between 3 and 24 months of age, indicating growth of the OB during ageing^28^. Generally, substantial growth of the OB in the opossum, a representative of marsupials and laboratory rodents, representative of eutherians occurs during ageing. Although the total volume of the OB increases during aging, the ability of perception of smells declines with age.

Several papers have studied the link between adult neurogenesis and age-related declination in the OB. In all examined species of mammals most of newly generated cells in the RMS migrate and are finally incorporated into the OB^29–32^. The vast majority of newly born neurons are located in the granule cell layer, making synapses with the mitral and tufted cells, while only few neurons are placed in the glomeruli layer, making synapses with the periglomerular cells^33^. Newborn neurons in both layers are later incorporated into local circuits and can be involved in analysis of olfactory information^34,35^. For many years, the role of adult neurogenesis is studied incessantly, and its contribution to plasticity of the brain is still reported^36^. Integration of newborn neurons into the OB circuits is required for olfactory learning^37,38^. Our results indicate that young opossums have more newly generated neurons in the OB than aged animals. Moreover, the lowest level of DCX, that is considered to be a specific marker for immature neurons, was detected in aged female opossums. Additionally, this group of opossums had the worst performance in the behavioral test. Three aged female opossums of 8, in spite of being hungry, were unable to find rewarded food during the first trial and needed twice more time to start digging during the test day. All these data allow us to suggest that new neurons of the OB are involved in analysis of novel odors. In agreement, previous studies showed that new born neurons of the OB in mice are responsive to novel odors^37^. Four months after proliferation and differentiation these new, but now mature neurons sustain this function, specifically they react to novel odors.

Many proteins are involved to participate during maturation process of neurons. Compelling evidence indicates that HuD is one of proteins that control neuronal differentiation during development, while in adult brain this protein is involved in plasticity^39^. HuD is a neuronal member of the Hu proteins family which are RNA-binding proteins. We found that HuD is located in upper layers including the mitral cell layer of the OB. Mitral cells receive afferent fibers from different olfactory cell types, therefore they are essential for analysis of olfactory signal at the OB^40^. The level of this protein is lower in the OB of young opossums than in aged animals. These results indicate that in the opossum, HuD is involved in the mechanism regulating negative olfactory responsiveness. Transgenic mice that overexpress HuD in neurons of the forebrain showed the poor performance in the Morris water maze and contextual fear conditioning test^14^. These data support our findings that there is an association between worse performance and increased level of HuD.

## Conclusion

Data presented in this work provide a new evidence that extension of the OB during aging does not contribute to better olfactory perception. Moreover, decreased rate of adult neurogenesis and increased level of HuD protein in the OB of aged female opossums are associated with the poor performance in olfactory-guided behavior.

## Methods

### Animals

Young (n = 14) and aged (n = 15) opossums of both sexes bred at the Nencki Institute of Experimental Biology colony were used. The housing facility was constantly monitored; temperature was kept at 26–28 °C, with a humidity of 40–70%, and daily cycle was regulated on 14/10 h (day/night). All efforts were made to minimize the number of animals used and the level of stress they endured. The study was carried out in compliance with the ARRIVE guidelines. The experimental procedures complied with the Polish Law on Experiments on Animals, which implements the European Council Directive and were approved by the 1st Warsaw Local Ethics Committee for Animal Experimentation (Permit Number: 272/2017).

### Olfactory discrimination test

To test learning and memory using olfactory cues in opossums the behavioral test was performed during 3 days. The main task was to determine two different odors, orange and lemon. A few drops of orange and lemon oils were applied in sawdust. A piece of the orange was buried in sawdust in the same place where drops of the orange oil was applied. 24 h before the test all opossums were restricted for food. Over the next 2 days all opossums received very limited amounts of food. Four trails were conducted during each training day. Behavioral tests were performed in a separate room. Opossums were placed in a bigger transparent cage (45 × 30 × 40 cm) that was filled with a thick layer of sawdust and 5–6 drops of lemon and orange oil were applied in sawdust in 2 different places. The location of these places in the cage was changed with each trail. To record opossums behavior in the cage, we used the PhenoRack system (ViewPoint Life Sciences, Inc.). Four cages, each was placed between CCD video cameras, were connected to a computer, and behavior of opossums was registered and analyzed during 5 min. Several measures including time spent to sniff in both places where odors were induced and latency to find food were calculated and analyzed.

On the third day, the test was performed without food reward. All parameters were measured that allow to estimate the level of learning using olfactory cues.

The body weight of opossums was measured twice, before and after the behavioral experiments.

### Tissue preparation

Half of the opossums from each group were perfused with saline followed by 4% paraformaldehyde in 0.1 M phosphate buffer (pH 7.4). Then the brains were removed, left in 4% paraformaldehyde solution, cryoprotected with 30% sucrose solution and cut into 40-μm coronal sections in a cryostat (Leica Biosystems). The brain sections were arranged in a series of ten. Two series were collected on slides and used for Nissl staining, while remaining series were collected in an antifreeze solution and stored at – 20 °C.

The remaining half of the opossums were sacrificed and their brains were isolated and weighed. Then, the OB were separated on ice, weighed and stored at − 80 °C.

### Nissl staining

Slides with brain sections of the OB were immersed for 30 min in a solution containing 50% chloroform and 50% ethyl alcohol for degreasing. Then the slides were passed through a series of ethanol (100%, 70%, and 50%) and washed in distilled water. The next step was incubation of slides in 0.33% cresyl violet acetate solution for 10 min. Subsequently, the slides were washed in distilled water and incubated in 50% ethanol, 70% ethanol with 10% acetic acid and 100% ethanol. The brain sections were degreased with xylene and cover-slipped with the mounting medium.

### Immunohistochemistry and estimating the number of labeled cells

Immunofluorescent staining was performed on free-floating brain sections. Brain sections were incubated for 2 h with 10% NGS and 1% bovine serum albumin in phosphate-buffered saline (PBS). Next, the sections were incubated in primary antibodies, rabbit anti-doublecortin (1:500, Cell Signaling) or mouse anti-HuD (1:100, Santa Cruz) overnight. The secondary antibodies, goat anti-rabbit 568 (1:500, AlexaFluor Invitrogene) or goat anti-mouse 488 (1:500, AlexaFluor Invitrogene) were used. Finally, the sections were mounted on slides and cover-slipped with a 60% glycerol solution in PBS.

To estimate the number of labeled cells in the OB, the representative sections were chosen from each group of opossums. Four selected areas corresponding medial, lateral, dorsal and ventral sides of each section (Supplementary Fig. S1 and Fig. S2) were imaged using a confocal microscope (Zeiss). We found that HuD and DCX neurons were located in different layers of the OB in the opossum. Therefore, the number of HuD cells was counted in the glomerular layer, external plexiform layer and mitral cell layer (Supplementary Fig. S22), while DCX cells were mainly analyzed in the granule cell layer and the external plexiform layer (Supplementary Fig. S1). The number of HuD and DCX labeled cells was counted using Image Fiji software that allows to count cells in the total depth of 40-μm sections. Afterwards, the density of labeled cells/mm^2^ was calculated. Analysis were made from comparable sections between 4 groups of animals. This manual counting method was adapted from Li et al.^41^.

### Western blot

The brain was homogenized with a hand held homogenizer in lysis buffer containing protease inhibitors (Roche). The samples were centrifuged at 14,000 rpm for 45 min at 4 °C, followed by incubation with NP 40 detergents (Fluka) and sodium dodecyl sulfate (SDS, Sigma). Next, the supernatant with proteins was collected. The protein samples (30 μg/lane) were separated by electrophoresis in 15% polyacrylamide gel and transferred onto a nitrocellulose membrane for 1.5 h at 100 mA at 4 °C. The blots were incubated in 5% skimmed milk powder dissolved in Tris-buffered saline with 0.2% Tween 20 for 1 h 30 min and incubated overnight at 4 °C with rabbit anti-doublecortin (1:1,000, Cell Signaling) or mouse anti-HuD (1:200, Santa Cruz). Afterwards, the blots were incubated with secondary antibody conjugated with horseradish peroxidase. The goat anti-rabbit (1:7,000, BioRad Laboratories) or goat anti- mouse (1:7,000, Millipore) secondary antibody was used. For visualization of proteins, WesternBright ECL HRP substrate (Advansta) was applied.

As a loading control TUJ protein was used (Supplementary Fig. S1a and Fig. S2a). After blocking, the blots were incubated with rabbit anti-TUJ antibody (1:10,000, Sigma) and goat anti-mouse secondary antibody (1:7,000, Millipore). Negative controls were performed without the primary or secondary antibody (Supplementary Fig. S1b and Fig. S2b). Positive controls were carried out using mouse tissue from the olfactory bulb (Supplementary Fig. S1b and Fig. S2b)^42,43^.

### Data analysis and statistics

Brain and the OB weights were analyzed using two way repeated measures analysis of variance (ANOVA) followed by the post hoc (GraphPad Prism). The same statistical tests were used to analyzed behavioral data and the level of proteins. Differences were considered significant for *p* < 0.05.

Images from Nissl-stained OB were obtained using a Nikon Eclipse 90i microscope connected to a computer with Neurolucida software (MBF Bioscience). Pictures from immunofluorescent sections were captured and analyzed using a confocal laser microscope (Zeiss). Western blots were scanned in a G:BOX Chemi XT4 equipped with a camera (Syngene) and the intensity of labeled bands was measured using GeneSys (Syngene) software.

## Supplementary information

Supplementary figures.
