# Supplementary material for: Impaired olfactory neurogenesis affects the performance of olfactory-guided behavior in aged female opossums
Source: Sci Rep. 2021 Feb 24;11:4418. doi: 10.1038/s41598-021-83834-5 (PMC7904797; doi:10.1038/s41598-021-83834-5)
Supplement: Supplementary file 1 — Supplementary figures. [file 41598_2021_83834_MOESM1_ESM.pdf]

Impaired olfactory neurogenesis affects the performance of olfactory-guided behavior in aged female opossums

Beata Tepper[1], Paulina Koguc-Sobolewska[1], Katarzyna Jaslan[1], Krzysztof Turlejski[2], Katarzyna Bartkowska[1], Ruzanna Djavadian[1*]

[1]Laboratory of Calcium Binding Proteins, Nencki Institute of Experimental Biology Polish Academy of Sciences, Warsaw, Poland
[2]Faculty of Biology and Environmental Sciences, Cardinal Stefan Wyszynski University in Warsaw, Warsaw, Poland

*** Correspondence:**

Ruzanna Djavadian

r.djavadian@nencki.edu.pl

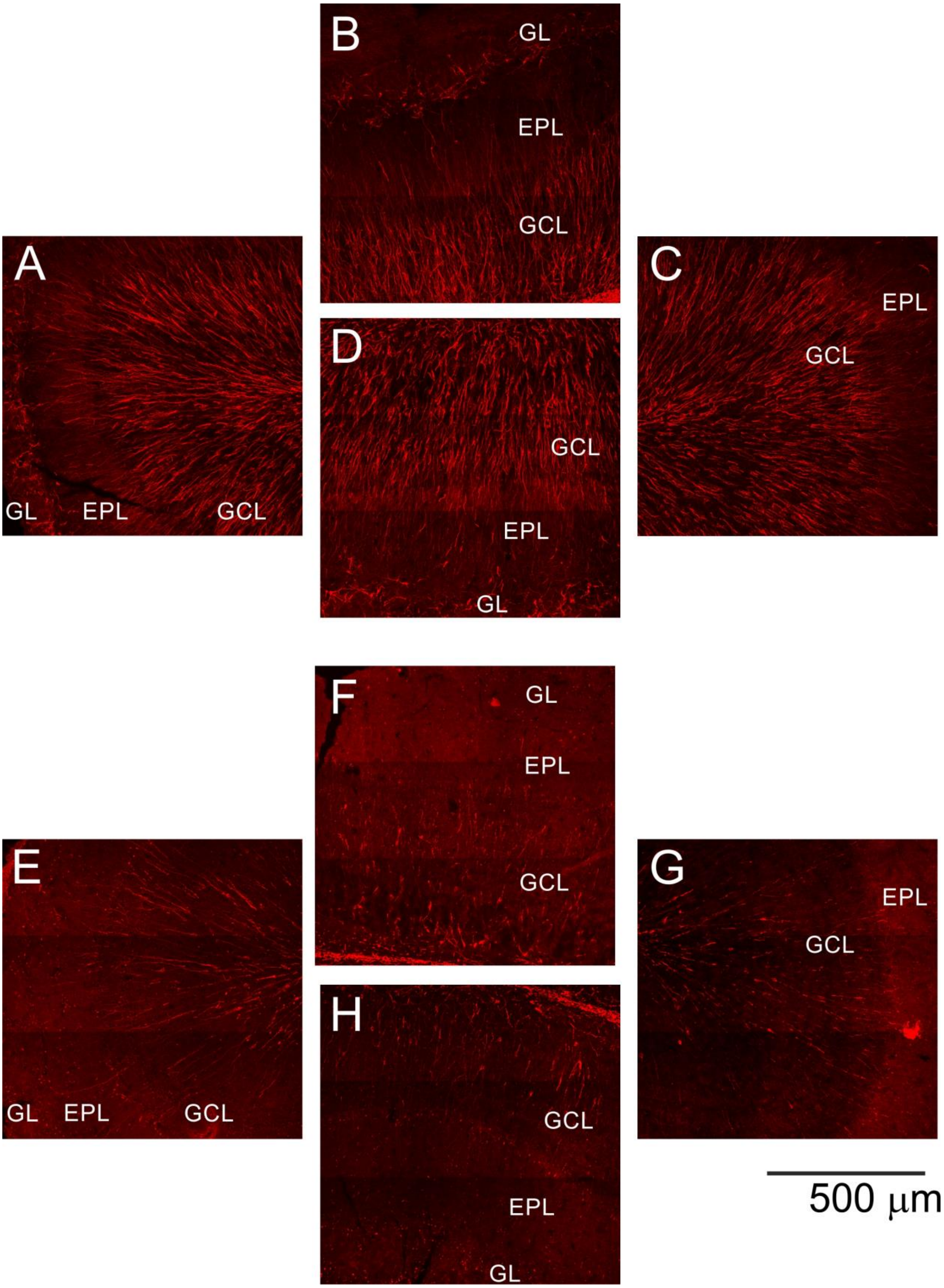

Supplementary Figure S1. Four selected areas of each section were imaged using a confocal microscope and DCX labeled cells were analyzed using Image Fiji software. (A-D) Images from one brain section of a young opossum. (E-H) Images from one brain section of an aged opossum.

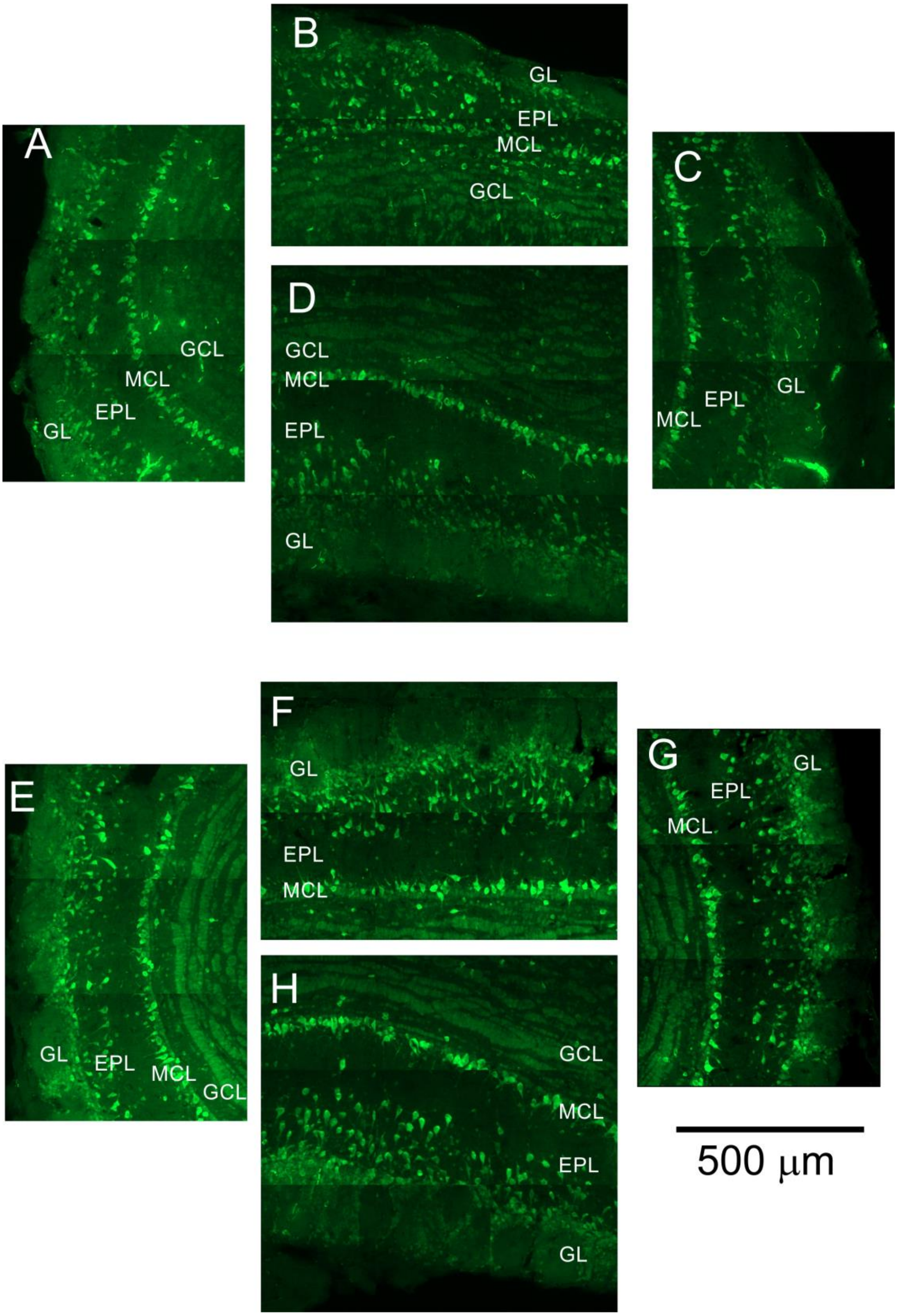

Supplementary Figure S2. Four selected areas of each coronal brain section were imaged using a confocal microscope and HuD labeled cells were analyzed using Image Fiji software. (A-D) Images from one brain section of a young opossum. (E-H) Images from one brain section of an aged opossum.

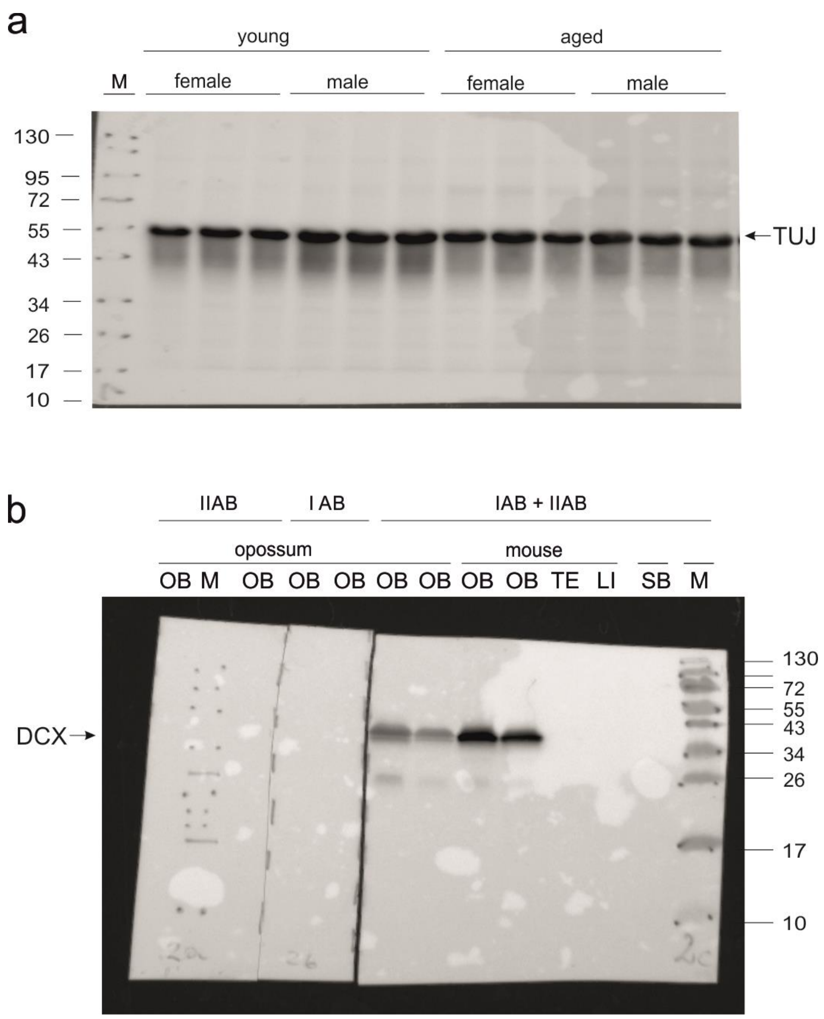

Supplementary Figure S3. (**a**) Full-length Western blot for TUJ protein that was presented in Fig. 5a. (**b**) Negative controls were performed without secondary (IIAB) or primary (IAB) antibodies or using sample buffer (SB), while positive controls were performed using opossum and mouse olfactory bulb (OB) tissues. Additionally, tissue homogenates of mouse testis (TE) and liver (LI) were used. M, marker.

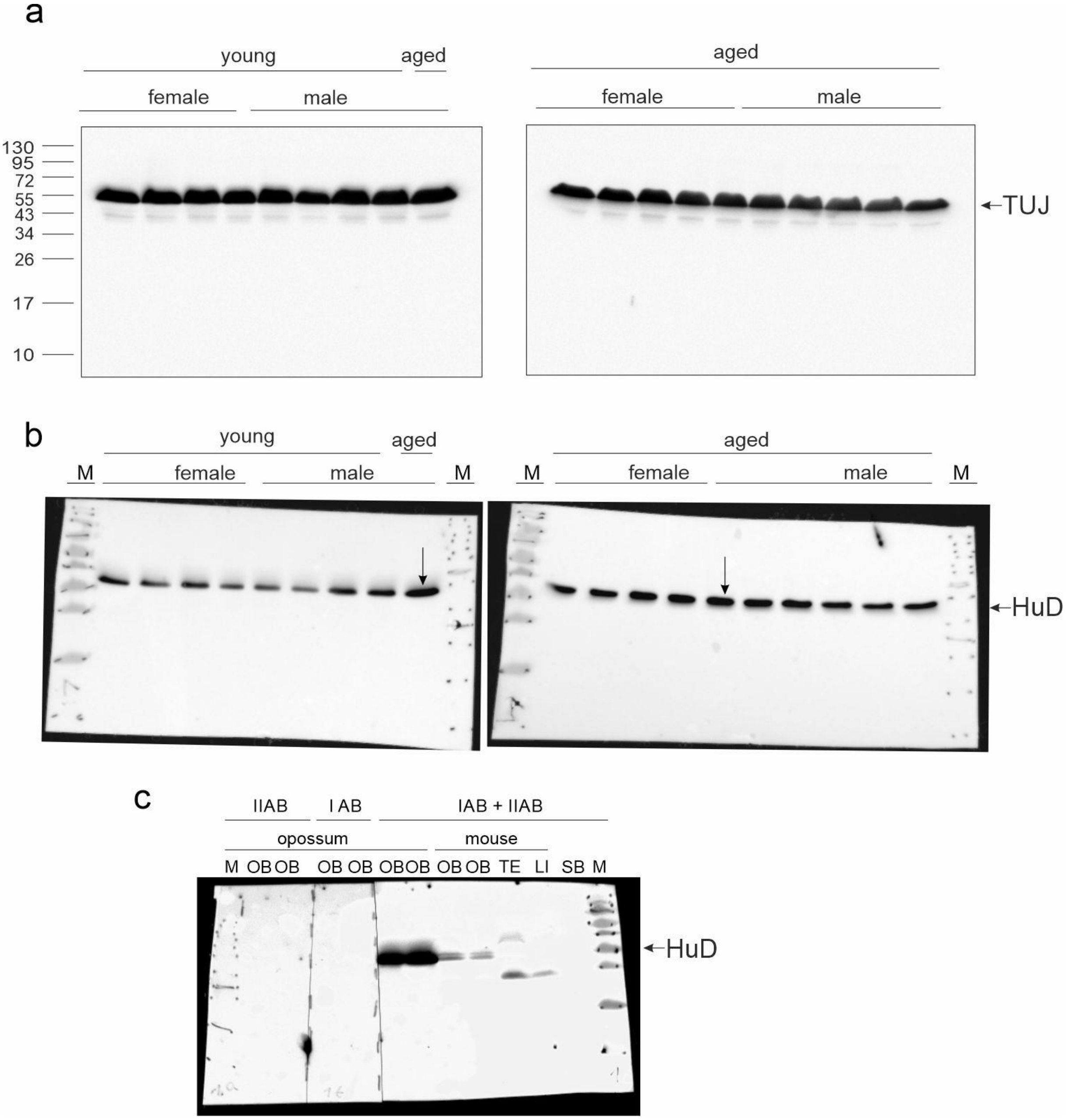

Supplementary Figure S4. (**a**) Full-length Western blots for TUJ protein that were presented in Fig. 7a. (**b**) We compared 2 Western blots using samples from the same opossum in both blots and found identical labeled band pattern of HuD protein. The arrows show these labeled bands. (**c**) Negative controls were performed without secondary (IIAB) or primary (IAB) antibodies or using sample buffer (SB), while positive controls were performed using opossum (aged) and mouse (young) olfactory bulb (OB) tissues. Additionally, homogenates of mouse testis (TE), liver (LI) were used. M, marker.
